# Incidence of oral cancer due to tobacco and alcohol use

**DOI:** 10.6026/9732063002001650

**Published:** 2024-11-30

**Authors:** Ashutosh Pandey, Imran Md., Anjana Bharti, Satya Prakash, Sweety Kumari, Sumit Verma, Manoj Mittal

**Affiliations:** 1 Department of Oral Medicine and Radiology & Department of Dentistry, Anugrah Narayan Magadh Medical College and Hospital, Gaya, Bihar, India; 2 Department of Dentistry, Patna Medical College and Hospital, Patna, Bihar, India; 3 Private Consultant, Buddha Dental Hospital, Patna, Bihar, India; 4 Department of Oral Medicine and Radiology, Buddha Institute of Dental Sciences and Hospital, Patna, Bihar, India; 5 Department of Conservative Dentistry and Endodontics; ECHS Polyclinic Khagariya, Bihar, India; 6 Department of Oral & Maxillofacial Surgery, Dr. B.R. Ambedkar Institute Of Dental Sciences & Hospital, Patna, Bihar, India; 7 Department of Periodontology, Bhabha College of Dental Sciences, Bhopal, Madhya Pradesh, India

**Keywords:** Oral cancer, tobacco, alcohol retrospective analysis

## Abstract

This study aims to evaluate the trends in oral cancer incidence in relation to tobacco and alcohol use, analyzing clinical data from the past three years to uncover patterns and potential correlations. A retrospective analysis was conducted on clinical data from 200 patients diagnosed with oral cancer within the last three years. Information regarding their tobacco and alcohol consumption was gathered and analyzed to identify trends and correlations with oral cancer incidence. The findings highlight the urgent need for public health strategies focused on reducing tobacco and alcohol use to lower the risk of oral cancer.

## Background:

Oral cancer remains a significant global health challenge, with incidence and mortality rates varying across regions [1]. The etiology of oral cancer is multifactorial, with tobacco and alcohol consumption being the predominant risk factors [2, 3]. Over the past decades, numerous studies have firmly established the association between these lifestyle factors and oral cancer development [4]. Despite advancements in medical research and public health initiatives, the incidence of oral cancer continues to rise in many parts of the world, emphasizing the need for a detailed analysis of recent trends and contributing factors [5]. This retrospective study aims to analyze oral cancer incidence trends over the past three years, focusing on the correlation between tobacco and alcohol use. By reviewing clinical data from patients diagnosed with oral cancer, the study seeks to provide insights into the current epidemiological landscape and highlight areas for potential public health interventions [6]. Oral cancer, predominantly consisting of squamous cell carcinomas, contributes significantly to global cancer-related morbidity and mortality [7]. According to the World Health Organization (WHO), oral cancer ranks among the top ten most common cancers worldwide [8]. The disease burden is disproportionately higher in developing countries, where tobacco and alcohol consumption is widespread [9]. The carcinogenic effects of tobacco and alcohol are well-documented. Tobacco smoke contains numerous harmful chemicals, including polycyclic aromatic hydrocarbons and nitrosamines, which induce genetic mutations and promote carcinogenesis [10]. Alcohol, acting as a solvent, enhances the penetration of carcinogens into the oral mucosa, leading to cellular damage and increasing cancer risk [4]. While overall cancer rates have declined in some regions due to improved screening and preventive measures, trends in oral cancer have been inconsistent [1]. In areas where tobacco and alcohol consumption remains high, there has been a marked increase in oral cancer cases [2]. Analysing these trends is essential for developing effective public health strategies [5, 6]. Given the significant role of tobacco and alcohol in the etiology of oral cancer, this study aims to provide a comprehensive analysis of oral cancer incidence trends over the past three years about these risk factors [3, 6]. By utilizing clinical data from a substantial patient cohort, the research seeks to identify patterns and correlations that could inform future prevention and intervention efforts [4]. This study's findings are expected to contribute to the existing knowledge on oral cancer epidemiology and provide evidence-based recommendations for public health policy [9]. By emphasizing the impact of tobacco and alcohol on oral cancer incidence, the research underscores the need for targeted interventions to reduce these modifiable risk factors [10].

## Methods and Materials:

## Study design:

This study employed a retrospective design, utilizing clinical data from patients diagnosed with oral cancer over the past three years. Approval for the study was obtained from the institutional review board, ensuring adherence to ethical standards and maintaining patient confidentiality throughout the research process.

## Sample size:

A total of 200 patients diagnosed with oral cancer between 2020 and 2023 were included in the study. The sample size was determined based on the availability of detailed clinical data and robust statistical analysis needed to identify meaningful trends and correlations.

## Data collection:

Data were extracted from medical records, including demographic variables (age, gender and ethnicity), clinical characteristics (tumor location, stage and histopathology) and lifestyle factors (tobacco and alcohol use). Tobacco use was classified into three categories: current users, former users and never users. Alcohol consumption was categorized as regular drinkers, occasional drinkers and non-drinkers.

## Statistical analysis:

Descriptive statistics were used to summarize the demographic and clinical characteristics of the patient cohort. Chi-square tests and logistic regression analyses were conducted to evaluate the association between tobacco and alcohol use and oral cancer incidence. Time-series analysis assessed trends in oral cancer incidence over the three-year study period.

## Results:

## Demographic and clinical characteristics:

The study included 200 patients diagnosed with oral cancer over the past three years. The mean age of the patients was 56 years, with a male-to-female ratio of 3:1. The majority of the study population (65%) were current tobacco users, 25% were former users and 10% had never used tobacco. In terms of alcohol consumption, 40% of patients were regular drinkers, 30% were occasional drinkers and 30% were non-drinkers (Table 1).

## Trends in oral cancer incidence:

A notable increase in oral cancer incidence was observed over the three-year study period, particularly among current tobacco users and regular alcohol drinkers (Figure 1 see PDF). The incidence rate among current tobacco users increased by 15%, while the incidence among regular alcohol drinkers rose by 10%. These findings suggest a strong association between these lifestyle factors and the rising trend in oral cancer cases. Chi-square tests revealed a statistically significant association between tobacco use and oral cancer incidence (p < 0.01), as well as between alcohol use and oral cancer incidence (p < 0.05). Logistic regression analysis further indicated that current tobacco users had an odds ratio (OR) of 2.5 (95% CI: 1.8-3.5) for developing oral cancer, while regular alcohol drinkers had an OR of 1.8 (95% CI: 1.2-2.8) (Table 2 and Table 3). These results confirm a strong correlation between tobacco and alcohol use and the increased incidence of oral cancer over the past three years.

## Discussion:

The results of this study underscore the substantial role that tobacco and alcohol consumption play in the rising incidence of oral cancer. Consistent with prior research, our findings revealed that current tobacco users have significantly higher odds of developing oral cancer, with an odds ratio (OR) of 2.5. At the same time, regular alcohol drinkers showed an OR of 1.8 [4, 6]. This is in line with previous epidemiological studies, which have established tobacco and alcohol as major risk factors for oral cancer, acting synergistically to elevate the risk of carcinogenesis [2, 7]. The observed increase in oral cancer incidence among current tobacco users (15%) and regular alcohol drinkers (10%) over the past three years is a concerning trend. This finding highlights the on-going challenge of reducing tobacco and alcohol use despite existing public health campaigns aimed at these behaviors [8]. In particular, developing countries with high rates of substance use are likely to experience a disproportionate burden of oral cancer [9]. Our study strongly advocates for enhanced public health policies targeting the reduction of tobacco and alcohol consumption. Effective interventions such as smoking cessation programs, regulation of alcohol availability and mass media campaigns aimed at raising awareness about the carcinogenic effects of these substances are critical [10]. Furthermore, policy measures such as increased taxation on tobacco and alcohol products and legislative restrictions on their marketing could also reduce consumption rates [3, 9]. Given the growing incidence of oral cancer associated with these risk factors, comprehensive public health strategies are essential for mitigating the disease's burden. While this study provides valuable insights into the correlation between lifestyle factors and oral cancer incidence, further research is warranted to explore the long-term effects of public health interventions on disease trends. Studies assessing the efficacy of smoking cessation and alcohol reduction programs on oral cancer rates over extended periods could inform policy decisions [5]. Additionally, future research should examine genetic predispositions and environmental exposures that contribute to oral cancer risk, which may help identify at-risk populations and enable more personalized prevention strategies [6].

Understanding these multifactorial influences is crucial for developing a holistic approach to reducing oral cancer incidence globally. Moreover, the role of social determinants of health in influencing tobacco and alcohol use cannot be overlooked. Factors such as socioeconomic status, education level and access to healthcare significantly impact an individual's likelihood of engaging in these behaviors. Populations in low-income and rural areas often have limited access to smoking cessation programs, alcohol counselling and cancer screening services, which exacerbates the disparities in oral cancer outcomes [11]. Addressing these inequalities by improving access to preventive services and education on the risks associated with tobacco and alcohol use should be a key component of future public health efforts. In addition to direct interventions targeting tobacco and alcohol use, there is a growing need for broader cancer prevention strategies that incorporate early detection and treatment of oral cancer. Screening programs, particularly in high-risk populations, can lead to earlier diagnosis, improved survival rates and lower healthcare costs [12]. Given the slow progression of oral cancer and its often asymptomatic nature in the early stages, regular screenings can play a vital role in reducing mortality. Public health systems should consider integrating oral cancer screening into routine care, especially in areas with high tobacco and alcohol consumption rates. Lastly, the implementation of educational programs in schools and communities that focus on the dangers of tobacco and alcohol use from a young age can foster long-term behavioral change. Early intervention is critical in preventing the initiation of these risky behaviors. Health literacy initiatives that provide clear information on the links between lifestyle factors and cancer risk, combined with supportive environments that promote healthy alternatives to smoking and drinking, can contribute to reducing the incidence of oral cancer over time [13, 14-15]. Public health campaigns must continuously evolve, utilizing modern communication platforms to reach younger audiences and high-risk groups effectively.

## Conclusion:

In conclusion, our findings reinforce the need for targeted public health interventions focusing on tobacco and alcohol control to address the rising incidence of oral cancer. Implementing evidence-based strategies could significantly reduce the risk of oral cancer and improve overall population health outcomes.

## Figures and Tables

**Table 1:** Demographic and clinical characteristics of the study population

**Characteristics**	**Number**
Age (Mean ± SD)	56
Male	150
Female	50
Current Tobacco Users	130
Former Tobacco Users	50
Never Users	20
Regular Drinkers	80
Occasional Drinkers	60
Non - Drinkers	60

**Table 2:** Correlation between tobacco use and oral cancer incidence

**Tobacco Users**	**Oral Cancer Incidence**
Current Users	130
Former Users	50
Never Users	20

**Table 3:** Correlation between alcohol use and oral cancer incidence

**Alcohol Users**	**Oral Cancer Incidence**
Regular Drinkers	80
Occasional Drinkers	60
Non - Drinkers	60
